# Soybean root elongation in relation to water and mechanical stresses in intact and disturbed structures of an Oxisol

**DOI:** 10.3389/fpls.2026.1805154

**Published:** 2026-04-14

**Authors:** John Kennedy dos Santos, Luiz Henrique Quecine Grande, Lucas Henrique Amaro da Silva, Matheus Batista Néri Pereira, Anaila Amaral de Alencar, Renato Paiva de Lima, Murilo dos Santos Vianna, Julio Cezar Franchini, Alvadi Antonio Balbinot Junior, Henrique Debiasi, Moacir Tuzzin de Moraes

**Affiliations:** 1 Department of Soil Science, Luiz de Queiroz College of Agriculture, University of São Paulo, Piracicaba, São Paulo, Brazil; 2 Center for Carbon Research in Tropical Agriculture (CCARBON), University of São Paulo, Piracicaba, São Paulo, Brazil; 3 Department of Water and Soil, School of Agricultural Engineering, University of Campinas (UNICAMP), Campinas, São Paulo, Brazil; 4 Institut für Bio- und Geowissenschaften: Agrosphäre (IBG-3), Forschungszentrum Jülich GmbH, Jülich, Germany; 5 Department of Soil and Crop Management, Embrapa Soybean, Brazilian Agricultural Research Corporation - Embrapa, Londrina, Parana, Brazil; 6 Embrapa Wheat, Embrapa, Passo Fundo, Rio Grande do Sul, Brazil

**Keywords:** *Glycine max* L., physical limitations, root elongation rate, saturation degree, semi-empirical model, soil penetration resistance

## Abstract

**Introduction:**

Process-based root growth simulation models detail soil-root interactions, but do not incorporate the role of soil structure dynamics. Our objective was to parameterize the soybean root elongation under mechanical and hydric stress levels for soybean roots grown in soil with preserved (Field) and packed structure (Packed).

**Methods:**

In a long-term field experiment under the no-tillage system, soil samples from an Oxisol with preserved and non-preserved structures were used to evaluate soybean root growth responses. Soil samples with non-preserved structure were reconstructed with under six bulk densities (1.02; 1.10; 1.18; 1.26; 1.34; 1.42 Mg m^-3^) considering the field range of degrees of compactness and used for soybean cultivation. Soil penetration resistance (mechanical stress), soil water content, soil porosity and degree of soil water saturation (hydric stress) were evaluated. Root elongation rates of soybean seedlings were measured. The soybean relative root elongation was the response variable used in the model, assessed by combining the effects of water and mechanical stress on root elongation, allowing for a qualitative comparison of soil structures (Field and Packed).

**Results:**

Field structure exhibited superior performance under conditions of high physical constraints, particularly in scenarios of elevated soil penetration resistance. An increase in soil penetration resistance from 1 MPa to 3.5 MPa resulted in a 52% reduction in relative root elongation under Field structure conditions, whereas in packed samples this reduction was more pronounced, reaching 74%. Under conditions of low soil penetration resistance (0.5 MPa) and a water saturation degree of 60%, relative soybean root elongation in packed samples was 29% higher than in field structure. However, the relative elongation between both structural conditions became similar as soil penetration resistance increased to 1.3 MPa. Furthermore, under near-saturation conditions (i.e., 90%), soybean root elongation in field structure was nearly three times higher than that estimated in packed samples.

**Discussion:**

Soil structure drives biophysical soil-root interactions, mitigating mechanical (soil penetration resistance) and hydric (degree of saturation) stresses on soybean root elongation under no-tillage, confirming our hypothesis via a semi-empirical model. Field structure under no-tillage facilitates root elongation through pore network, enhancing tolerance to water excess and deficit versus packed conditions. Degree of saturation provides practical water stress assessment, linked to soil penetration resistance via Busscher equation, capturing non-linear interactions for soybean root elongation responses to soil physical stresses. Incorporating soil structure effects into simulation models enhances root growth prediction, supporting effective agricultural practices under soil conservation management systems.

## Introduction

During growth within the soil, plant roots experience different levels of hydric stress, expressed as poor aeration or water deficit, mechanical impedance, and extreme temperatures (Bengough et al., 2011; Moraes et al., 2025), comprising a dynamic root–soil interaction along the soil profile (Landl et al., 2019; Bertollo et al., 2021). However, these biophysical interactions are driven by soil structure, especially by pore space characteristics (Jin et al., 2013). Recent efforts have focused on revealing the role of soil structure in mitigating soil physical stress for root elongation (Xiong et al., 2022; Islam et al., 2021), but little progress has been made toward quantifying this soil structure effect on root–soil interactions. Qin et al. (2025) proposed a novel empirical model to estimate root elongation as a function of soil penetration resistance and macropore volume, based on undisturbed soil samples. However, hydric stress was not considered, which represents a fundamental physical stressor (Moraes and Gusmão, 2021), especially due to its high variability throughout the crop cycle and along the soil profile. Furthermore, in accordance with the important role of soil structure highlighted in the comprehensive review by Gao et al. (2016a), this structure-driven root elongation can be evidenced by modeling root elongation under distinct structural conditions (e.g., undisturbed and packed samples), which remains lacking.

Although it has been highlighted the biopores potential to sustain root growth in structured soils (Behrend et al., 2025), there is still a lack of quantitative evidence on to what extent biopores or continuous pores can reduce mechanical and water-related stresses, thereby promoting root elongation in crops under no-tillage systems. Process-based models have significantly contributed to advances in understanding soil biophysical dynamics (Mulazzani et al., 2022), particularly the soil–root interactions (Schnepf et al., 2020; Seidel et al., 2022). Nevertheless, improvements in soil process modeling are needed (Jarvis et al., 2024) to better quantify and predict the environmental factors associated with crop development. The model proposed by Moraes et al. (2018) provides a mechanistic approach to represent root elongation rates as a function of mechanical and water limitations, enabling the simulation of crop root growth while accounting for soil physical constraints and varying weather conditions.

There is a gap in the literature regarding the root growth simulation for agricultural crops, especially when considering the physical limitations imposed by soil conditions (Schnepf et al., 2020; Mimault et al., 2023). Most of the existing models partially address mechanical and water stress factors, without fully integrating the effects of soil structure, such as the presence of biopores and continuous pores, on root elongation (Landl et al., 2019; Phalempin et al., 2022; Giuliani et al., 2024). Therefore, it is possible to enhance soil mechanical stability (Moraes et al., 2017, 2019) and crop resilience (Balbinot Junior et al., 2024) by no-tillage systems that preserve soil structure (Moraes et al., 2024; Grande et al., 2026). To achieve this, modeling of the physical stresses impact on root elongation is necessary for key grain-producing crops such as soybean, which are still not fully parameterized in root architecture and growth simulation algorithms (Schnepf et al., 2020; Seidel et al., 2022; Giraud et al., 2023).

A mechanistic model for predicting the soil physical stresses that affect root elongation (Moraes et al., 2018; Seidel et al., 2022) may incorporate the impacts of soil structure and biopores on the crops root elongation. In this way, the model can be used as a tool to predict the influence of soil physical quality on plant growth. Accordingly, the question to be addressed is how to express, in physical-mathematical terms, the effect of biopores and continuous pores in alleviating mechanical and water stress on growth of taproot systems (e.g., soybean). We hypothesize that a semi-empirical models can incorporate the effects of soil structure in mitigating the main impact of mechanical and water stress on the soybean root elongation, by the relationship between root elongation, soil mechanical impedance and degree of water saturation under different soil structural conditions. Our objective was to define the parameters of the root relative root elongation as a function of mechanical and hydric stress, for soybean grown in soil with a preserved and non-preserved structure.

## Material and methods

### Models experimental design

The parameterization of total physical stress models for soybean root elongation was developed by combining soil water and mechanical stress levels under different soil structural conditions in soil PVC columns (16.5 cm height and 5 cm inner diameter), consisted of: (i) soil samples with preserved structure, derived from a no-tillage system (Field) and (ii) soil samples with packed structure (Packed), representing the immediate impact of soil disturbance after tillage. The experiment was carried out in a completely randomized design with 22 replicates per matric potential in Field soil and 18 replicates per matric potential in Packed soil. Total soil physical stress affecting soybean root elongation comprises both mechanical and water stress. Mechanical stress was represented by soil penetration resistances. The stablished bulk density intervals (mechanical impedance levels) for packed soil were determined based on the observed variability of the bulk density in field plots (accordingly to preserved structure samples), aiming to create a similar and comparable mechanical impedance range between both structural conditions (i. e., Field and Packed). Water stress was represented by the soil degree of water saturation, determined by the hydrostatic equilibrium of soil samples at soil water matric potentials of -10, -60, -100, -1000, and -5000 hPa. A total of 200 soil samples were used to develop the models, including 110 samples (22 per matric potential) with preserved structure and 90 (18 per matric potential) with packed structure. The degree of water saturation was established as a hydric stress variable due to its straightforward and practical measure and relationship with the water-filled pores fraction.

### Experimental site and soil sampling

This study was based on a long-term field experiment, established in 2016 under the no-tillage system. The experiment was conducted at the experimental site of the National Soybean Research Center (Embrapa Soja), located in Londrina, Paraná, Brazil (23°11′ S, 51°11′ W) at an elevation of 630 m (Moraes et al., 2016). The regional climate is classified as humid subtropical (Cfa) according to the Köppen classification system, with a mean annual temperature of 21 °C (Alvares et al., 2013). The mean annual rainfall is 1622 mm (Alvares et al., 2013). The soil at the experimental site is classified as Rhodic Eutrudox according to the Soil Taxonomy system (Soil Survey Staff, 2022) and as Latossolo Vermelho Distroférrico in Brazilian Soil Classification System (Santos et al., 2018). The soil has a very clayey texture, with 755 g kg^-1^ clay, 178 g kg^-1^ silt, and 67 g kg^-1^ sand. The particle density in the 0–0.30 m layer is 2.96 Mg m^-3^. The maximum bulk density, determined by the Proctor test, is 1.53 Mg m^-3^ (Torres and Saraiva, 1999).

**Figure 1:**
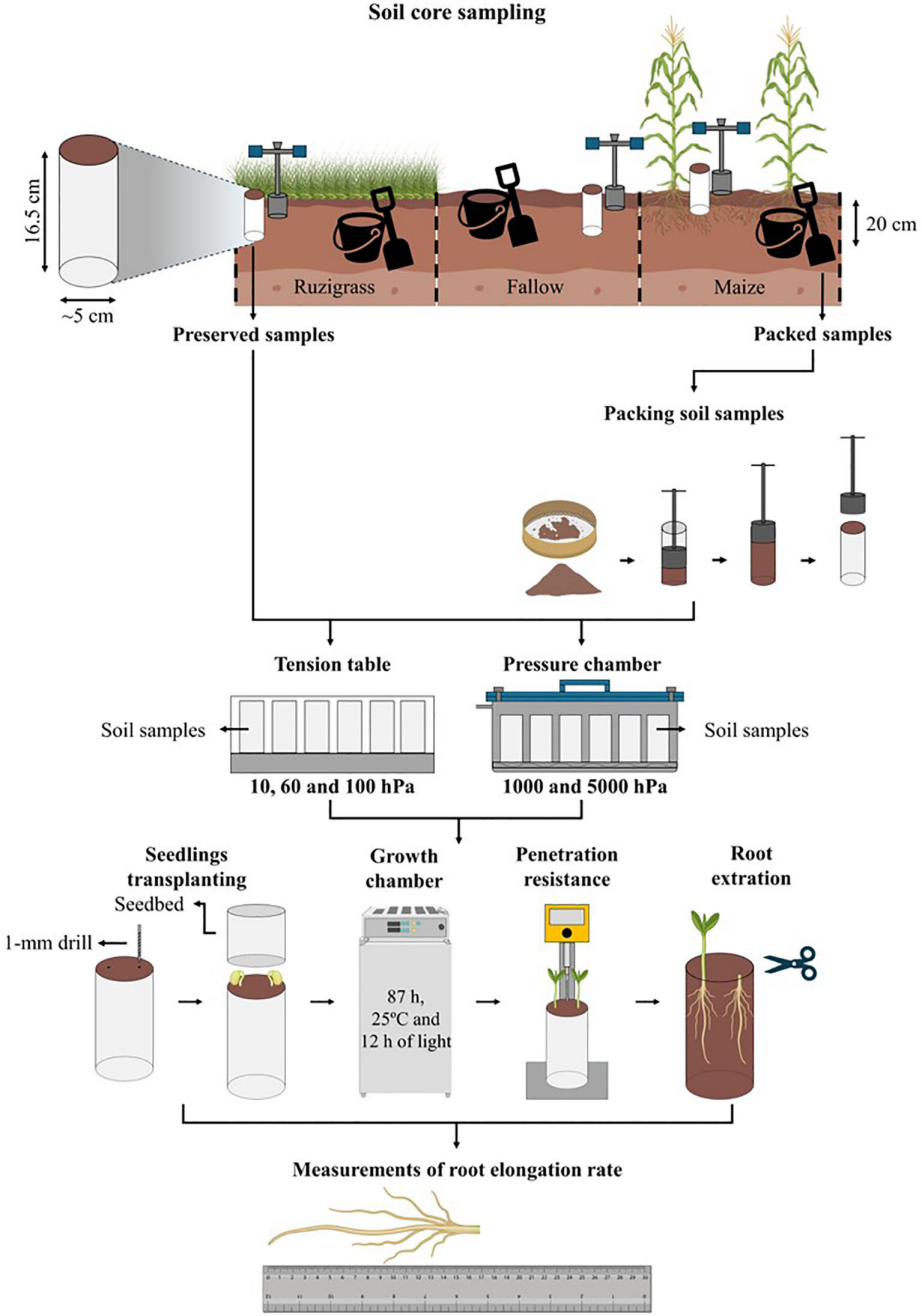
Scheme of utilized methodology to evaluate the soybean root elongation rate as a function of soil physical stress for each structure (i. e., Field and Packed).

Soil sampling was divided into preserved structure (i. e., Field) and non-preserved structure (i. e., Packed) (**Figure 1**). The soil samples were collected in September 2023 from three crop systems during the soybean off-season: (i) ruzigrass (*Urochloa ruziziensis*) without fertilizers used as cover crop, (ii) fallow, and (iii) maize (*Zea mays* L.) with topdressing nitrogen fertilizer (80 kg N ha^-1^). Soil samples from all three crop systems were collected during the same sampling period. Further details regarding the field experiment setup can be found in Balbinot Junior et al. (2024). It was collected 110 field samplest using an impact-type core sampler (Uhland), with a total height of 18.5 cm, adjusted to fit PVC cylinders measuring 16.5 cm height and 5 cm inner diameter. Samples were collected at a depth of 0–20 cm from various points within the experimental area to obtain samples with a wide range of degrees of compactness. The samples with preserved soil structure were wrapped in plastic film, placed in appropriate boxes to prevent structural damage and moisture loss, and carefully transported to the laboratory.

Soil samples for structure packing were obtained at the same soil depth and points in the experimental area, following the same sampling procedure used for the preserved structure samples, but these were transported to the laboratory, where they were sieved through a 2 mm mesh to homogenize the material and stored in plastic bags, maintaining the water content close to the soil friability level. The preparation of different soil compaction levels by bulk densities (1.02; 1.10; 1.18; 1.26; 1.34; 1.42 Mg m^-3^) into the packed samples was based on the soil gravimetric water content, followed by the calculation of the dry soil mass required to achieve the target bulk densities, corresponding to the volume of the same PVC cylinders used for field sampling (298.6 cm^3^). This soil mass was divided into five equal parts, corresponding to the desired densities, and compacted in 3.3 cm layers inside the PVC cylinders using a manual compactor. The procedure was repeated for each target density, ensuring uniform compaction across the samples, totalizing 90 packed soil samples (6 compaction levels × 5 hydric stress levels).

### Soybean root elongation and physical conditions

Soybean seedlings (cultivar BRS 1061IPRO) were transplanted in each soil sample after its hydrostatic equilibrium at respective soil water matric potentials (i. e., -10, -60, -100, -1000 and -5000 hPa) to evaluate the root elongation rate as a function of soil physical stress levels and structure. Soybean seeds were selected and sterilized in a 0.2% calcium hypochlorite solution, washed with deionized water and germinated on specific paper (Germitest) for 32 hours in a germination chamber at 25 °C, with 80% relative humidity and a 12-hour photoperiod. Subsequently, two seedlings with radicle lengths between 5 and 10 mm were transplanted into each soil sample as sub-replicates, which were used to assess the average root elongation within the soil sample. The soil samples were previously saturated by capillarity, and the saturated soil mass was recorded, considering the cylinder mass. The samples were hydrostatically equilibrated at each matric water potential (18 and 22 soil samples for each matric water potential for Field and Packed soil, respectively), and the soil mass was recorded to verify water loss throughout the procedures, which was negligible after the root growth period.

To do this, 1-mm diameter and 10 mm deep holes were made in the soil surface with a spiral drill. The samples were then transferred to a growth chamber for 87 hours under controlled conditions (temperature at 25 °C, relative humidity between 60–80%, and a 12-hour photoperiod). Soil penetration resistance was measured up to 70 mm depth immediately after the growth period, using a bench penetrometer (CT3™ Texture Analyzer model), consisting of a metal rod with a conical tip, featuring a 30° semi-angle, 4 mm diameter, and a base area of 0.1256 cm^2^, operating at a constant penetration rate of 2 mm S^-1^. The roots were then carefully extracted from the cylinders and separated from the soil by washing with water using a 0.5-mm sieve and forceps for handling. Following washing, the roots were preserved in containers with 70% ethanol. The length of the primary root was measured to quantify the root elongation rate, calculated as the ratio of root length to development time (i.e., 87 hours) (Valentine et al., 2012; Bengough et al., 2016). The root elongation rate and the relative root elongation for each soil sample and treatment were assessed as an average of the two soybean seedlings.

Soil water content and dried-soil mass were determined using 40 g of soil collected from each sample, after the seedling growth phase and soil penetration resistance evaluation, and oven-dried in a forced-air circulation oven at 105 °C for 4 days. Soil bulk density (Mg m^-3^) was determined as the ratio between the dry soil mass and the cylinder volume. Total porosity (m^3^ m^-3^) was calculated as the difference between the mass of saturated soil and the mass of dry soil, divided by the sample volume. Gravimetric soil water content (kg kg^-1^) was determined as the ratio between the mass of water and the mass of dry soil, whereas the volumetric soil water content (m^3^ m^-3^) was obtained by dividing the volume of water by the sample volume. Air-filled porosity (m^3^ m^-3^) was determined as the difference between total porosity and volumetric soil water content. The degree of water saturation (S, %) was calculated as the ratio between volumetric water content (m^3^ m^-3^) and total porosity (m^3^ m^-3^).

### Data analysis

The models for calculated soil penetration resistance were generated by fitting the penetration resistance values to soil bulk density and water content (Equation 1) for each soil structure condition (S1), using a non-linear model described by Busscher (1990).

(1) $$Q_{p}\;=\;a\;\rho^{b}\theta^{c}\;$$

where, *Q_p_* is the calculated soil penetration resistance (MPa), *ρ* is the bulk density (Mg m^-3^), *θ* is the soil volumetric water content (m^3^ m^-3^), while *a*, *b* and *c* are model fitting parameters.

The dataset (200 soil samples; 110 samples for Field structure and 90 samples for packed soil) was previously fractionated into 70% used to model fitting (n = 69 for Field and n = 60 for Packed) and 30% used to assess its performance (n = 30 for Field and n = 25 for Packed). Statistical analysis involved the fitting of root elongation into non-linear regression for each structural condition (i. e., Field and Packed), which corresponded to different semi-empirical models (Equation 4). The model based on the relationship between soybean relative root elongation, calculated soil penetration resistance, and degree os water saturation was developed using a three-dimensional (3D) Gaussian function (Equation 2), due to its ability to describe complex non-linear relationships among these variables. The choice of this model was based on its flexibility in fitting experimental data, allowing for an efficient representation of the observed patterns.

(2) $$Re\left(Q_{p},\;S\right)\;=a\;exp\;\left(-\frac{1}{2}\left({\left(\frac{S-x_{0}}{b}\right)}^{2}+{\left(\frac{Q_{p}-y_{0}}{c}\right)}^{2}\right)\right)\;$$

where *Re* is the relative root elongation, *S* is the degree of water saturation (%), *Q_p_* is calculated soil penetration resistance (MPa), while *a*, *b*, *c*, *x_0_*, and *y_0_* are function empirical parameters. The data were analyzed by regression using the software SigmaPlot^®^ 14 (Systat Software Inc.).

The agreement between simulated and measured values was based on one-third of the dataset, evaluated using several performance indicators, including the root mean square error (RMSE) (Equation 3) (de Jong van Lier et al., 2008); the coefficient of residual mass (CRM) (Equation 4); the correlation coefficient (r) (Equation 5) (Bonfante et al., 2010); and the index of agreement (d) (Equation 6) (Casaroli et al., 2010). The root mean square error (RMSE) has an ideal minimum value near zero and is a metric that reflects model performance differences in quadratic terms, being particularly sensitive to outliers.

(3) $$RMSE=\;\sqrt{\frac{1}{n}\sum_{i=1}^{n}{\left(P_{i}-O_{i}\right)}^{2}}$$

The residual mass coefficient (CRM) ranges from −∞ to +∞, with zero being the optimal value. Positive values indicate a tendency of the model to underestimate predictions, while negative values suggest overestimation. When the CRM approaches zero, it indicates the absence of systematic bias.

(4) $$CRM=\;\frac{\sum_{i=1}^{n}O_{i}-\sum_{i=1}^{n}P_{i}}{\sum_{i=1}^{n}O_{i}}$$

The ideal value of the correlation coefficient (r) is 1, indicating a perfect correlation, while a value near zero suggests no correlation (Addiscott and Whitmore, 1987).

(5) $$r\;=\;\frac{\sigma OP}{\sigma O.\sigma P}\;$$

where σOP represents the covariance between measured and predicted data, and σO and σP correspond to the standard deviations of the measured and predicted values, respectively. The agreement Willmott index (d) is a dimensionless metric ranging from -1.0 to 1.0 and more accurately reflects model performance compared to other indices (Willmott et al., 2012).

(6) $$d=1-\;\frac{\sum_{i=1}^{n}(P_{i}-O_{i}{)}^{2}}{\sum_{i=1}^{n}(\left|P_{i}-\underline{O}\left|+\right|O_{i}-\underline{O}\right|{)}^{2}}\;$$

The Busscher non-linear model was fitted to the measured data using the routine “PROC NLIN” following the Gauss-Newton method from the Statistical Analysis System 9.4 – SAS (SAS Institute Inc, 2013), and the graphs plotted through the program SigmaPlot^®^12.5 (Systat software, Inc.). Analyses of variance and model performance parameters were developed in the R software (R core Team, 2026), whereas the parametrization of semi-empirical models was performed using the SigmaPlot^®^12.5.

## Results

### Root elongation rate and physical stress

**Figure 2:**
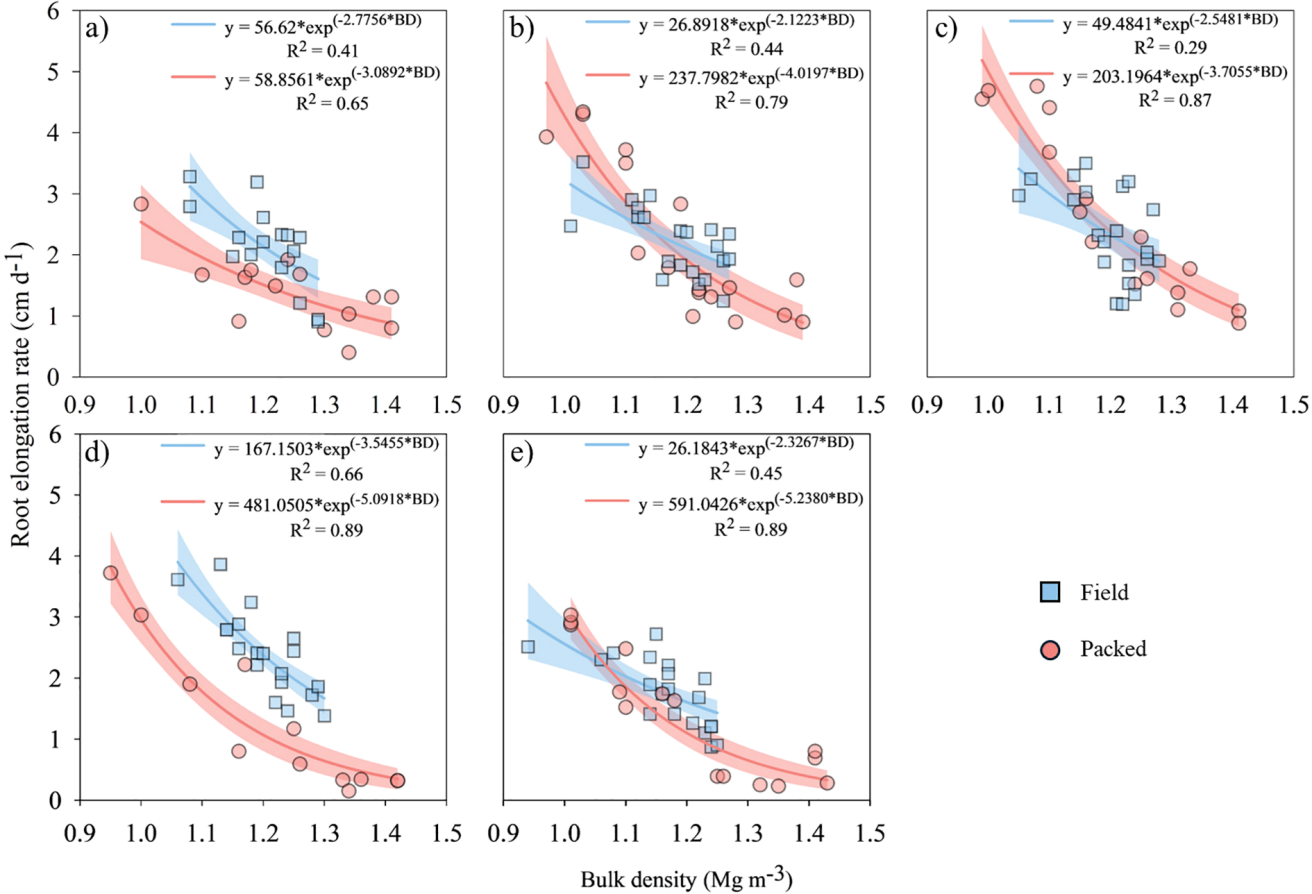
Soybean root elongation rate as a function of structural conditions (Field and Packed soil), bulk density (BD) at soil water matric potentials of -10 hPa **(a)**, -60 hPa **(b)**, -100 hPa **(c)**, -1000 hPa **(d)** and -5000 hPa **(e)**.

The elongation of soybean roots was influenced by both bulk density (or soil compactness) and soil water matric potential, primarily governed by soil structural conditions (**Figure 2**). The regression analyses revealed exponential relationships between root elongation rate and bulk density across all soil water matric potentials (-10 to -5000 hPa) (**Figure 2**). The packed structure exhibited stronger statistical fits, with coefficients of determination (R^2^) ranging from 0.65 to 0.89, whereas the field structure showed lower R^2^ values, between 0.29 and 0.66. These models demonstrated a more rapid decline in root elongation with increasing bulk density in the packed structure compared with the field structure, particularly under water-limited conditions (-1000 and -5000 hPa). At -10 hPa (**Figure 2a**), -1000 hPa (**Figure 2d**) and -5000 hPa (**Figure 2e**), the most pronounced differences were observed between packed and field structural conditions. In contrast, under conditions near field capacity (-60 hPa, **Figure 2b**; -100 hPa, **Figure 2c**), the regression curves for both structural conditions (Field and Packed) remained closely aligned throughout the bulk density range.

Under non-restrictive conditions, particularly at a soil water matric potential of -100 hPa and a bulk density of 1.00 Mg m^-3^ (**Figure 2c**), a root elongation rate of 3.89 cm d^-1^ was observed in the field soil structure, compared with 5.00 cm d^-1^ in the packed structure. However, under dry-soil conditions (-5000 hPa, **Figure 2e**) associated with a low bulk density of 1.10 Mg m^-3^, the root elongation rate decreased to 2.56 cm d^-1^ and 3.13 cm d^-1^ for field and packed conditions, respectively. This represented reductions of approximately 34% in the field soil structure and 37% in the packed structure when comparing the -5000 hPa (**Figure 2e**) results with the non-stressed -100 hPa condition (**Figure 2c**). In water-limited conditions, soil structure had a substantial effect on the soybean root elongation rate. For soybean grown at -5000 hPa, where the root elongation rate of soybean for the field structure was 1.01 cm d^-1^ compared with 0.38 cm d^-1^ in packed soil samples. Already at -1000 hPa under a low bulk density of 1.10 Mg m^-3^, the root elongation rate was 2.96 cm d^-1^ for field samples and 2.56 cm d^-1^ for packed soil samples (**Figure 2d**). Under severe soil compaction conditions (bulk density of 1.40 Mg m^-3^, at -1000 hPa), the root elongation rate decreased from 1.16 cm d^-1^ in field structures to 0.38 cm d^-1^ in packed soil samples.

### Effect of soil physical stress on soybean root diameter classes and root surface area

**Figure 3:**
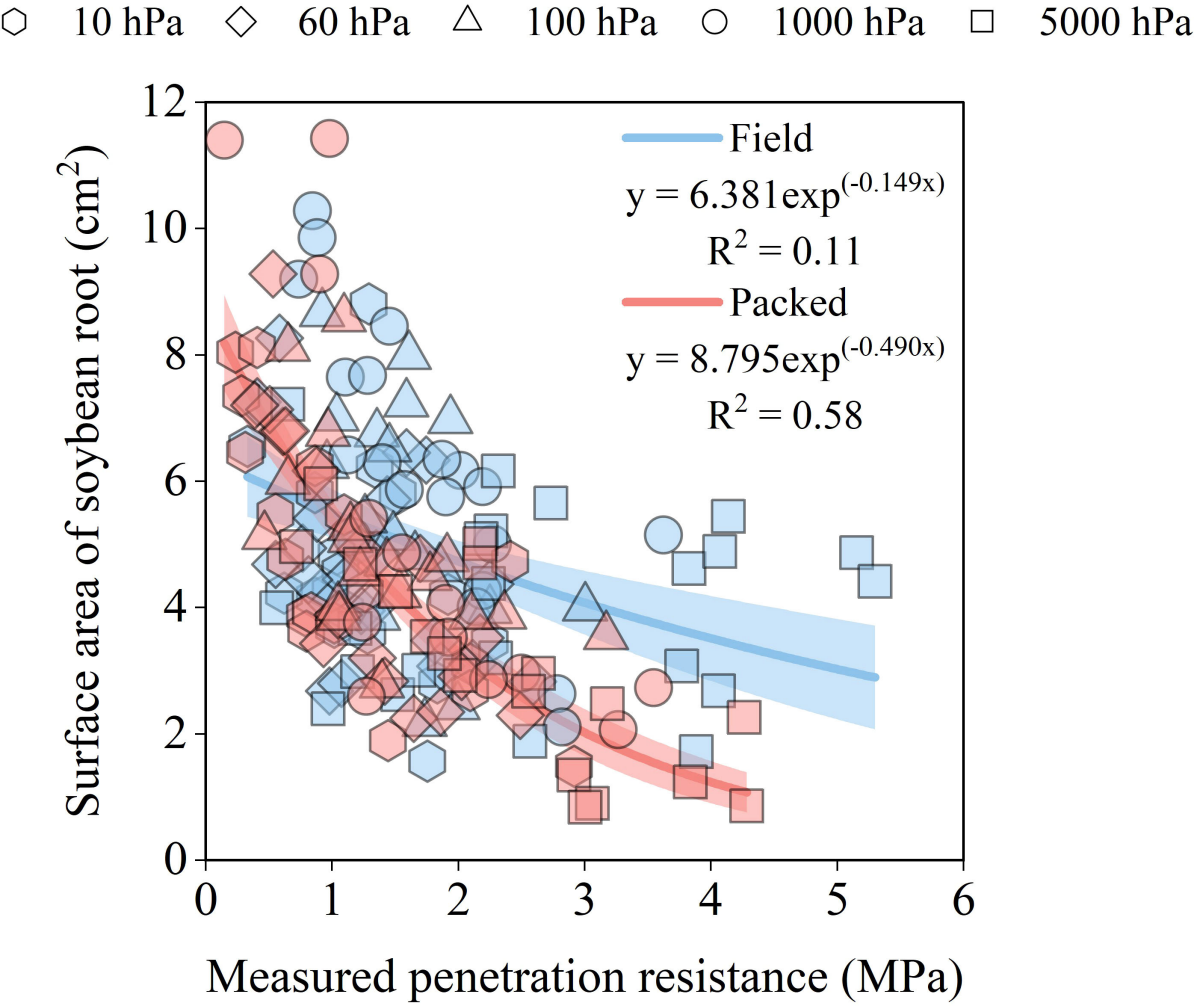
Relationship between soybean root surface area and measured soil penetration resistance in soils with field and packed structure. Points with different markers in the scatter plot represent the observed values from soil samples with Field and Packed structure equilibrated hydrostatically at soil water matric potentials of -10 hPa, -60 hPa, -100 hPa, -1000 hPa, and -5000 hPa. The lines represent the fitted regression models, and the shaded areas indicate the 90% confidence intervals of the fitted models.

**Figure 4:**
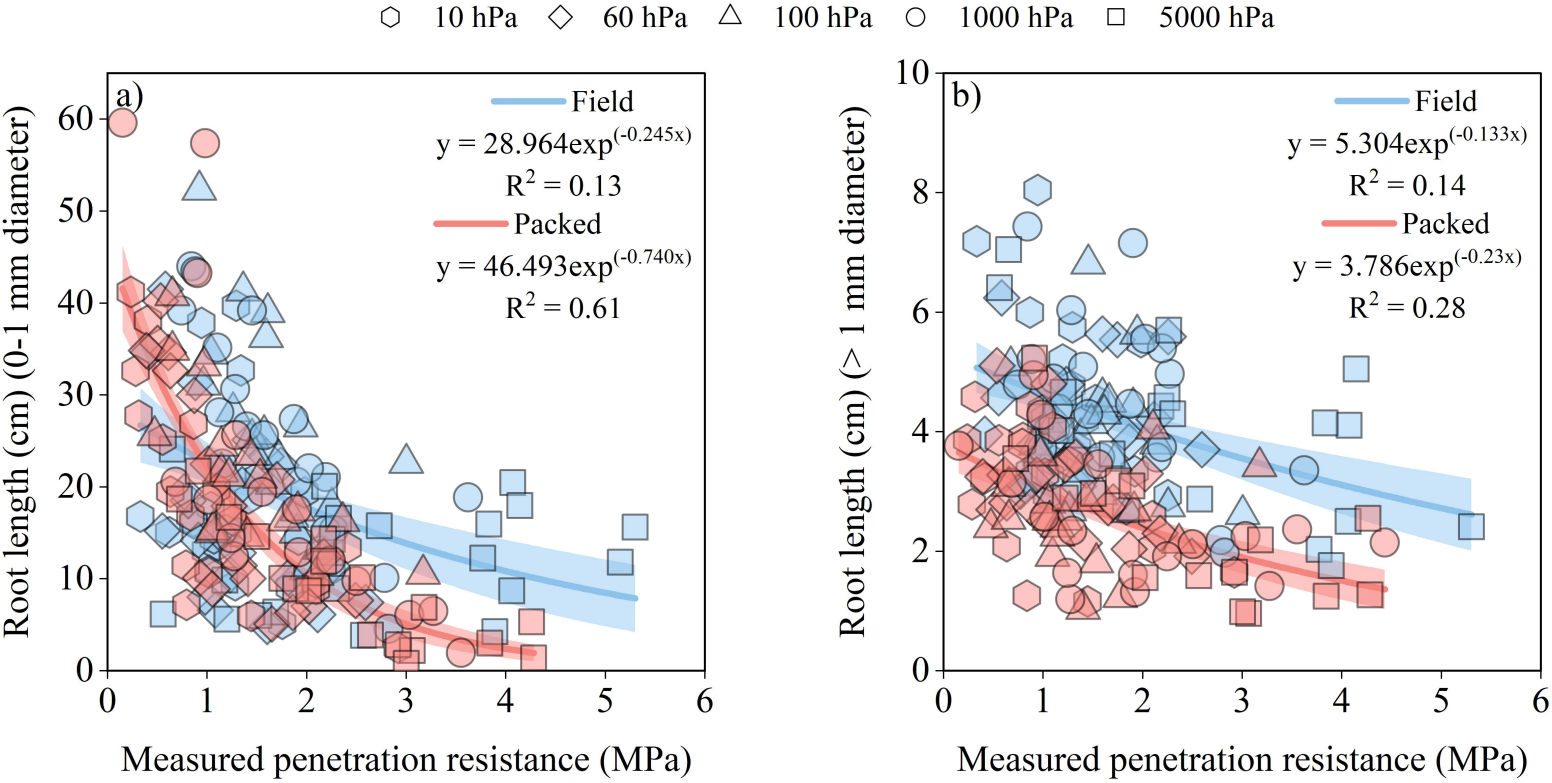
Impact of mechanical stress in soils with different structures (Field and Packed) on the root diameter classes of soybean seedlings: **(a)** absolute root length of roots with diameter ≤ 1 mm as a function of measured soil penetration resistance; **(b)** absolute root length of roots with diameter > 1 mm as a function of measured soil penetration resistance. Points with different markers in the scatter plot represent the observed values from soil samples with Field and Packed structure equilibrated hydrostatically at soil water matric potentials of -10 hPa, -60 hPa, -100 hPa, -1000 hPa, and -5000 hPa. The lines represent the fitted regression models, and the shaded areas indicate the 90% confidence intervals of the fitted models.

Soil mechanical and water stresses affected both root surface area (**Figure 3**) and root length across soybean root diameter classes (**Figure 4**). Root surface area declined with increasing measured soil penetration resistance in both structural conditions. Within the range of 0.5 to 4 MPa, root surface area decreased from 6.0 to 3.52 cm² in the Field structure and from 6.88 to 1.24 cm² in the Packed structure, corresponding to reductions of 41% and 82%, respectively (**Figure 3**). Under low measured soil penetration resistance (0.5 MPa), the Packed structure showed a root surface area 14% greater than that observed in the Field structure. However, as measured soil penetration resistance increased to 1 MPa, root surface area became slightly higher in the Field structure (2%) than in the Packed soil (**Figure 3**). The largest difference between structural conditions occurred at 4 MPa, where root surface area in the Field structure was 65% greater than in the Packed soil (**Figure 3**). Soil water potential also influenced root surface area. The highest mean values were observed at -1000 hPa, reaching 6.21 cm^2^ in the Field structure and 5.16 cm^2^ in the Packed soil (**Figure 3**). In contrast, the lowest mean values occurred at -5000 hPa, with root surface areas of 4.05 cm^2^ and 3.05 cm^2^ in the Field and Packed structures, respectively (**Figure 3**). These values correspond to reductions of 35% (Field) and 41% (Packed) in relation to the maximum surface area observed for each soil structure.

Root length responses varied between diameter classes and soil structures, being strongly affected by soil physical stress (**Figure 4**). The effect was more pronounced for fine roots (diameter ≤1 mm) (**Figure 4a**). Across the range of measured soil penetration resistance from 0.5 to 4 MPa, root length in this diameter class decreased by 58% in the Field structure (from 25.62 to 10.87 cm) and by 93% in the Packed structure (from 32.11 to 2.41 cm) (**Figure 4a**). Fine roots were also sensitive to soil water conditions. Under non-limiting water conditions (-100 hPa), the greatest mean root length was observed in both structures (26.51 cm in Field and 21.31 cm in Packed) (**Figure 4a**). Under severe water deficit (-5000 hPa), fine root length decreased by 54% in the Field structure and by 58% in the Packed structure (**Figure 4a**). In contrast, roots with diameter >1 mm were primarily affected by mechanical impedance (**Figure 4b**). Root length in this class decreased exponentially with increasing measured soil penetration resistance, with reductions of approximately 37% in the Field structure and 55% in the Packed structure within the range of 0.5 to 4 MPa (**Figure 4b**). The largest difference between soil structures occurred at 4 MPa, where root length in the Field structure was approximately twice that observed in the Packed soil (**Figure 4b**). Soil water conditions also affected the length of thicker roots. Under near-saturated conditions (-10 hPa), the highest mean root length values were observed in both soil structures (4.5 cm in Field and 3.1 cm in Packed) (**Figure 4b**). Under non-limiting water conditions (-100 hPa), mean root length decreased by 12% in the Field structure and by 18% in the Packed soil relative to -10 hPa (**Figure 4b**). Under severe water deficit (-5000 hPa), mean root length in the Field structure was approximately twice that observed in the Packed soil (**Figure 4b**).

### Modeling soybean root elongation and physical impedance

**Figure 5:**
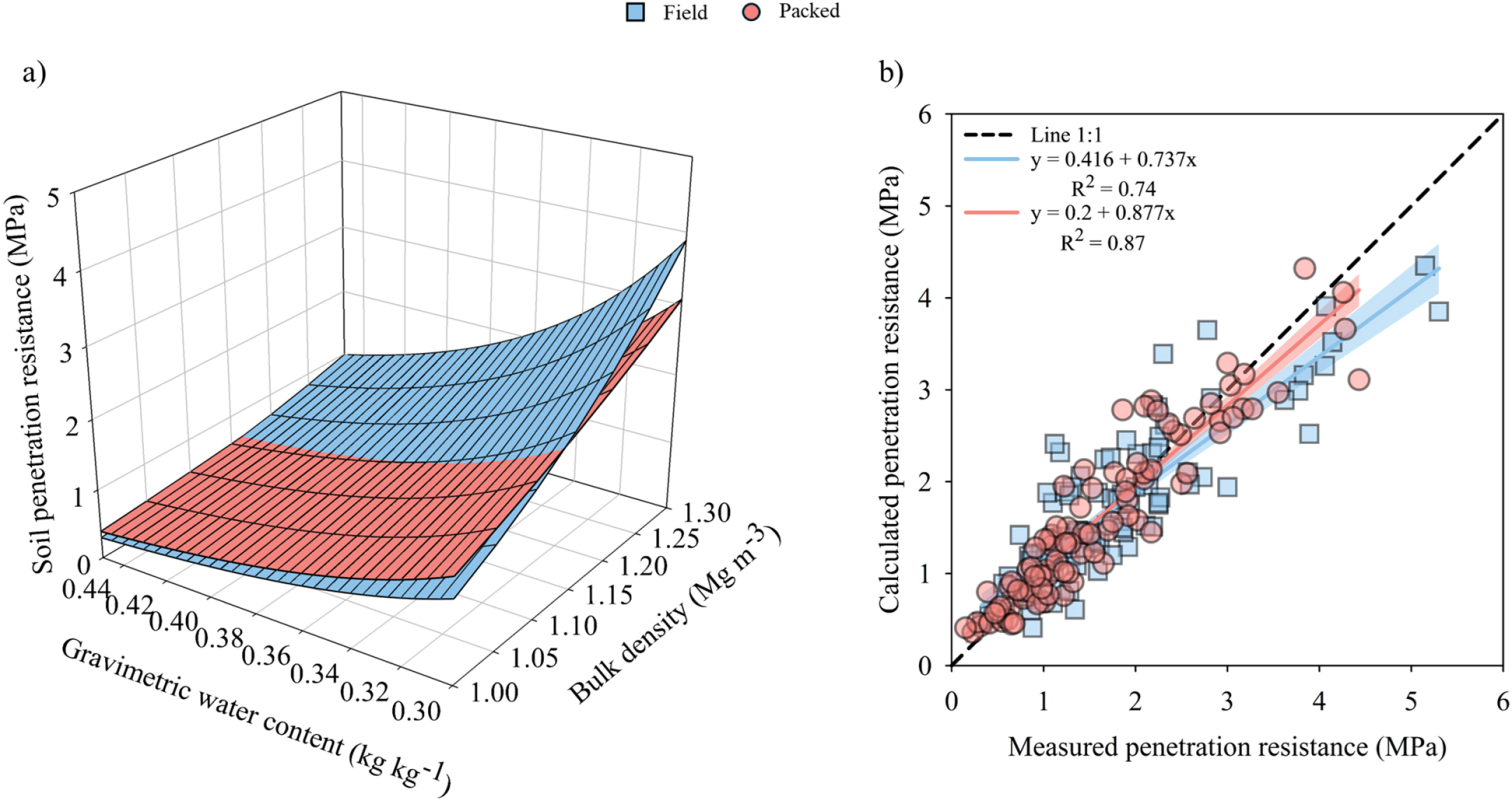
Soil penetration resistance model as a function of bulk density and gravimetric water content for a Rhodic Eutrudox with Field and Packed structure **(a)**, and calculated and measured values of soil penetration resistance for Field and Packed structure **(b)**. The blue and red bands indicate the 90% confidence interval.

Soil penetration resistance was acutely affected by bulk density and soil water content across the structural conditions (**Figure 5**). In both cases (i.e., Field and Packed), Buscher model performance was satisfactory, with determination coefficients of 0.97 and 0.95, respectively, indicating a high predictive capacity. Additionally, the root mean squared error (RMSE) was 0.50 MPa for the Field soil structure, and 0.35 MPa for the Packed structure (**Table 1**). The lowest calculated soil penetration resistance obtained was 0.29 MPa for Field soil structure and 0.38 MPa for Packed structure, associated with a soil bulk density of 1.0 Mg m^-3^ (the lowest value observed in the field) and a water content of 0.44 kg kg^-1^, close to soil saturation (**Figure 5a**). However, as soil bulk density increased to 1.3 Mg m^-3^ and soil water content decreased to 0.3 kg kg^-1^, that is, under conditions of high compactness and dry soil, calculated penetration resistance in the Packed structure reached 3.43 MPa, whereas in the Field soil structure it reached 4.35 MPa, a 27% higher value (**Figure 5a**). Thus, the bulk density increase and water content decrease resulted in an approximate 15-times increase in calculated penetration resistance in Field soil structure and 9-times increase in packed-structure condition, which represent a large mechanical impedance to soybean root elongation.

**Table 1:** Empirical parameters of the soil penetration resistance models for a Rhodic Eutrudox under field and packed structures.

Soil structure	Soil penetration resistance model^1^	Standard error for estimated parameters			R²	RMSE
		a	b	c		
Field	Q_p_ = 0.0201 BD^4.5602^ W^-3.3804^	0.0056	0.6398	0.2291	0.95	0.504
Packed	Q_p_ = 0.0286 BD^2.8134^ W^-3.2738^	0.0083	0.2603	0.2796	0.97	0.353

**^1^**Soil penetration resistance model in function of bulk density and water content (Q_p_ = a.BD^b^.W^c^); Q_p_ is the calculated soil penetration resistance (MPa); BD is the bulk density (Mg m^-3^); W is the soil gravimetric water content (m^3^ m^-3^); R^2^ = [1-(SQresidual/SQregression)]; RMSE: root mean square error (in MPa).

**Figure 6:**
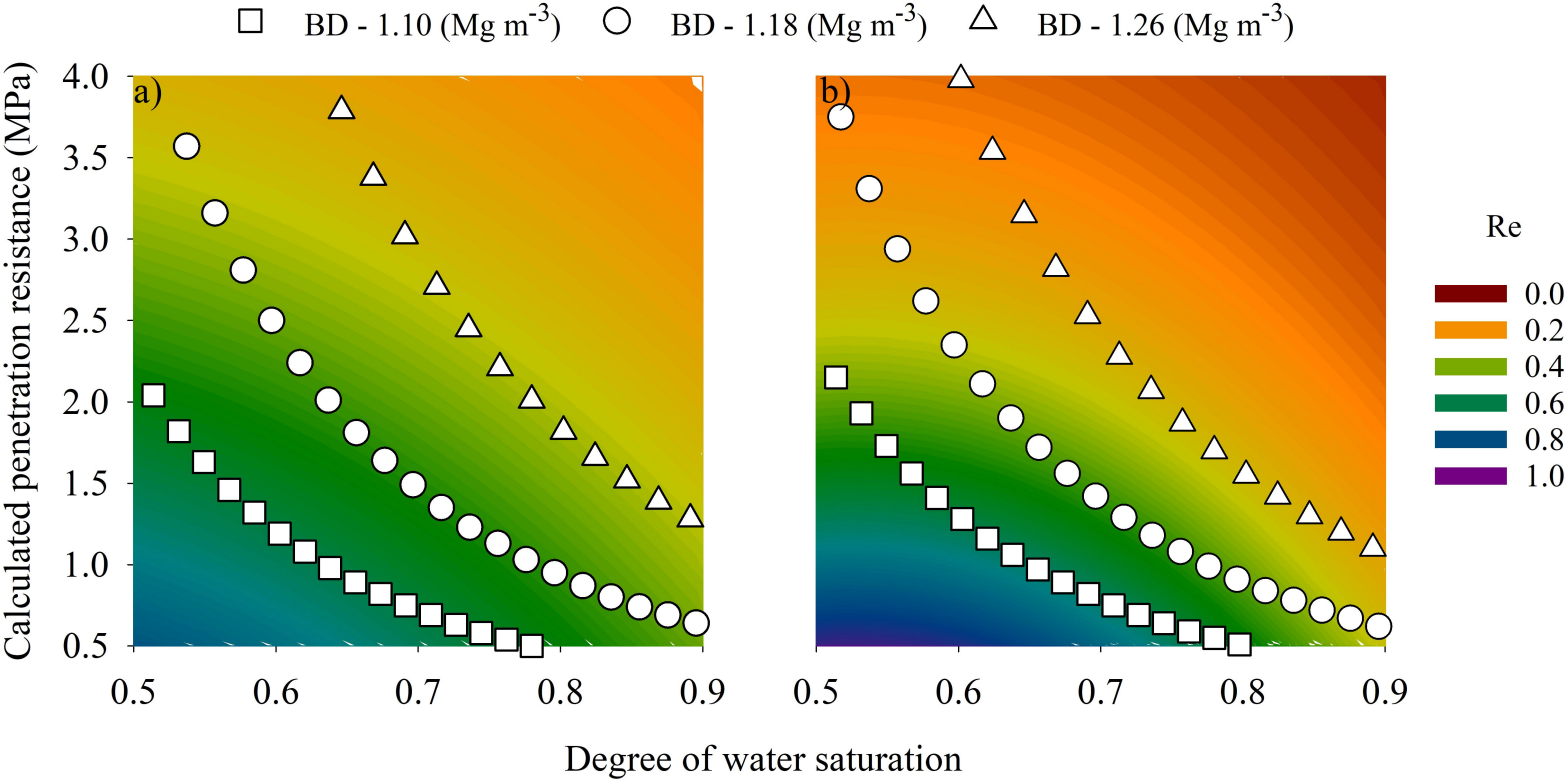
Estimated soybean relative root elongation (0-1) for field soil structure **(a)** and Packed soil **(b)** as a function of calculated soil penetration resistance and degree of water saturation in a Rhodic Eutrudox. The symbols for bulk densities (1.10 Mg m^-3^ (square), 1.18 Mg m^-3^ (circle) and 1.26 Mg m^-3^ (triangle)) indicate the relationship between calculated soil penetration resistance and degree of water saturation. Root elongation parameters are expressed as a relative index, ranging from 1 (maximum root elongation in blue color) to 0 (no root elongation in red color).

The comparison between predicted relative root elongation under different soil structural conditions revealed substantial differences between the developed models for preserved soil structure (Field model) and packed soil (Packed model) (**Table 2**). Overall, the estimated root elongation in the Field soil structure model was higher as mechanical stress increased (i.e., at higher penetration resistance levels) (**Figure 6**). On average, the structured soil model estimated a soybean relative root elongation 33% higher than those estimated for packed soil, with differences reaching up to 62% under combined high calculated soil penetration resistance and degree os water saturation (*Q_p_* = 4 MPa; *S* = 90%). Conversely, under conditions of low calculated soil penetration resistance (*Q_p_* = 0.5 MPa) and moderate water saturation (S = 50–70%), the Packed soil model slightly outperformed the structured model, estimating relative root elongation on average 16% higher. The major differences in the estimated root elongation between structural conditions were observed within the calculated soil penetration resistance range of 1.5 to 4.0 MPa, at a degree os water saturation of 90%, where the effect of soil structure on the mechanical and hydric stress mitigation was most pronounced.

**Table 2:** Soybean root elongation model with fitted parameters for each semi-empirical model.

Treatments	Soybean root elongation model^1^	R^2^
Field	$srf\left(Q_{P},\;S\right)\;=\;2.520\;exp^{\left(-\frac{1}{2}*\left({\left(\frac{\;S\;-\;33.763}{47.583}\right)}^{2}+\;{\left(\frac{QP\;+\;8.659}{6.116}\right)}^{2}\right)\right)}$	0.96
Packed	$srf\left(Q_{P},\;S\right)\;=\;994.145\;exp^{\left(-\frac{1}{2}*\left({\left(\frac{S\;-\;52.327}{25.88}\right)}^{2}+\;{\left(\frac{QP\;+\;27.157}{7.398}\right)}^{2}\right)\right)}$	0.92

^1^Semi-empirical model for soybean root elongation: $srf\left(QP,\;S\right)\;=a\;exp\left(-\frac{1}{2}\left({\left(\frac{S-x_{0}}{b}\right)}^{2}+{\left(\frac{Q_{P}-y_{0}}{c}\right)}^{2}\right)\right)$; A, B, C, X_0_ and Y_0_ are empirical parameters adjusted by the 3D Gaussian model (Equation 2) for structured soil and packed soil samples. Q_p_ is calculated soil penetration resistance; S is degree of water saturation; R^2^ = [1−(SQ_error_/SQ_total_)].

According to observed soybean elongation rate, both generated semi-empirical models showed a significant reduction in root elongation as a function of increasing calculated soil penetration resistance and near-saturation conditions (**Figure 6**). The maximum soybean relative root elongation was attained under low soil mechanical impedance (≤0.3 MPa) and a degree of soil water saturation close to 60%, equivalent to a soil water matric potential between -60 and -100 hPa. Similarly, within the low calculated soil penetration resistance range (0.3 MPa), soybean relative root elongation decreased from 0.74 to 0.43 in Field soil structure (**Figure 6a**) and from 0.97 to 0.35 in Packed structure (**Figure 6b**) as soil water saturation increased from 60% to 90%, corresponding to a reduction of 42% and 64%, respectively. The increase in calculated soil penetration resistance from 1 MPa to 3.5 MPa (soil water saturation of 60%) implied in a reduction in relative root elongation from 0.62 to 0.30 in Field structure conditions, representing a 52% decrease, whereas in Packed structure this reduction was more pronounced (74%), with the relative elongation varying from 0.68 to 0.18.

When grown under low bulk density of 1.10 Mg m^-3^ (i.e., degree of soil compactness of 72%, calculated soil penetration resistance of 0.5 MPa and a degree of water saturation of 60%) the soybean relative elongation in Packed structure (0.89; 4.29 cm d^-1^) was numerically 29% higher compared to that estimated in Field soil structure (0.69; 3.33 cm d^-1^), although the relative elongation between the two structural conditions became similar as calculated soil penetration resistance increased to 1.3 MPa. Under a bulk density of 1.26 Mg m^-3^ (i.e., 82% compactness and a calculated soil penetration resistance of 2.5 MPa) the relative elongation in Field soil structure (0.51; 2.44 cm d^-1^) was 1.6 times greater than the estimated in Packed soil (0.31; 1.49 cm d^-1^), becoming more pronounced as calculated penetration resistance increased to 4 MPa, resulting in elongation 2.1 times greater in Field soil structure. Furthermore, under near-saturation conditions (i.e., 90%), soybean root elongation in Field soil structure (0.13; 0.65 cm d^-1^) was almost 3 times higher compared with the estimated in Packed soil (0.05; 0.23 cm d^-1^).

**Figure 7:**
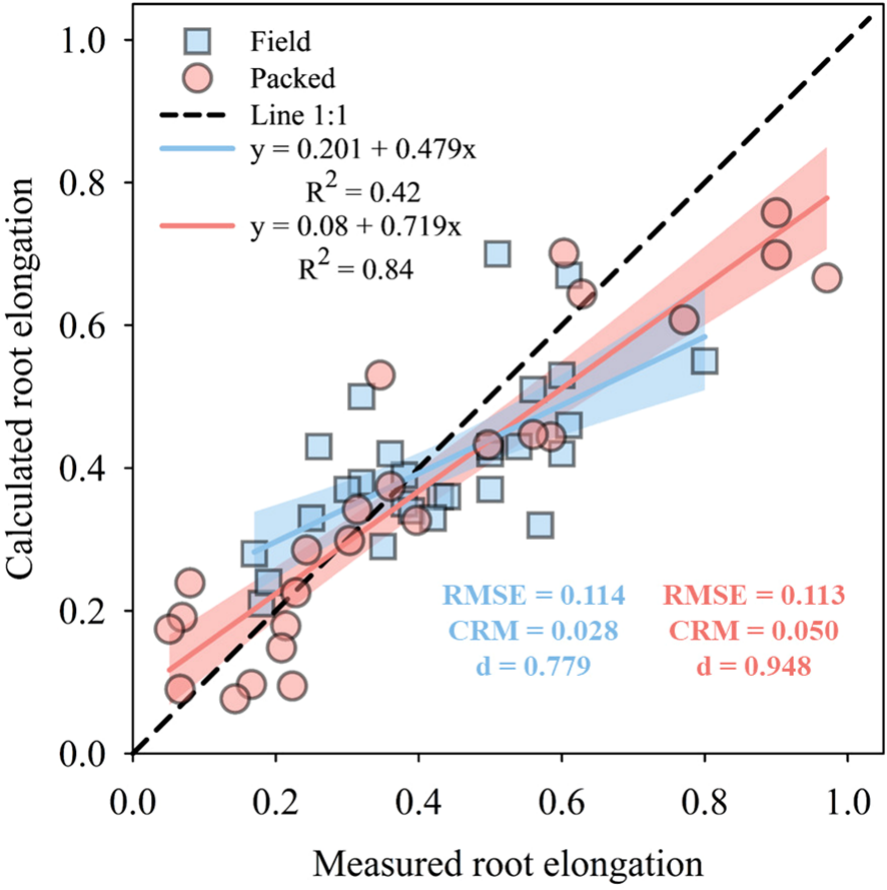
Estimated and observed values of soybean root elongation for field structure and packed soil. In the measured data, root elongation is represented by the relative root elongation, while in the simulated data, it is expressed by the relative root elongation function derived from the relationship between root elongation rate, calculated soil penetration resistance, and degree of water saturation using the three-dimensional (3D) Gaussian model. The blue and red bands indicate 90% confidence intervals.

The model for Field soil structure (**Figure 7**) demonstrated satisfactory performance in predicting the soybean relative root elongation. The root means square error (RMSE) of 0.114 was within acceptable limits, indicating a good overall accuracy. The residual mass coefficient (CRM) of 0.028 shows that the model does not present significant bias, while the moderate correlation (r = 0.66) suggests that the model effectively captures data trends. Furthermore, the high agreement index (d = 0.779) indicates strong predictive capability. Similarly, in the soil with Packed structure (**Figure 7**), the root means square error (RMSE) of 0.113 was slightly higher compared to Field soil structure, indicating that the predictions for the Packed soil present a slightly greater error, although still of good performance. The residual mass coefficient (CRM) of 0.050 indicates a negligible positive bias, without significantly impacting the model accuracy. The correlation (r) of 0.93 reveals a strong relationship between the observed and estimated root elongation values, demonstrating a good fit and solid model performance for the Packed soil. The high agreement index (d) of 0.948 reflects strong predictive accuracy, indicating that the model for the Packed soil is highly reliable.

## Discussion

We investigated the role of soil structure for driving the biophysical soil-root interaction and its capacity to mitigate the negative impact of soil mechanical resistance and degree of water saturation on soybean root elongation, markedly under a no-tillage system. The data confirm our hypothesis, revealing that soil structure plays a crucial role in mitigating mechanical and hydric stresses, allowing this effect to be parameterized (e. g., **Figure 6**). This structure-driven interaction not only facilitates root development but also enhances the plant capability to access water, expanding the optimal range for root growth even under conditions of water excess or deficit.

The parameterization, which incorporated the effect of soil structure into the relative root elongation function by the relationship between root elongation, soil penetration resistance, and degree of water saturation using the three-dimensional (3D) Gaussian model, proved particularly useful to understand the impact of mechanical and hydric stress on soybean root elongation. Moraes et al. (2020) reported the impact of soil physical stresses on soybean root elongation by root growth modeling, based on a mechanistic framework. That soil physical stress from Moraes et al. (2018) was also incorporated into a dynamic functional model (Seidel et al., 2022) coupling SIMPLACE and CRootBox models to estimate the mechanical and hydric stress for wheat and barley. However, the soil structure effect on driving the interaction between root elongation and soil physical stress has not yet been considered (Jin et al., 2013; Xiong et al., 2022), despite the soil structure role highlighted in our study. Recently Qin et al. (2025) propose an empirical model that includes the effects of macropores and soil penetration resistance on maize root elongation rate. The elongation rate of maize was reduced due to increased soil penetration resistance and increased air-filled porosity (Qin et al., 2025), in accordance with the pronunced root elongation decreasing observed in our study as soil penetration resistance increased. Structure-dependent root elongation parameterization may be important for improving the predictive capacity of process-based models (Schnepf et al., 2018b; Giraud et al., 2023), particularly because root elongation strongly influences other root growth and architectural parameters (i.e., root length density and root volume) (Schnepf et al., 2018a), which are fundamental for modeling root water uptake within the soil–plant–atmosphere continuum (Melo et al., 2025). Furthermore, understanding the main biophysical mechanisms of root–soil interactions may contribute to management strategies based on root growth and deepening in the soil, especially when considering stress scenarios driven by climate change in agroecosystems (George et al., 2024).

In this study, we advanced the piecewise stress reduction function of Moraes et al. (2018), which applied simplistic Feddes-based hydric stress limits (h1-h4 parameters for linear reduction of root elongation), with a semi-empirical model coupling mechanical (soil penetration resistance) and hydric (degree of water saturation) stresses on soybean root elongation. This addresses oversimplification risks in soil-plant-atmosphere interactions (de Melo and de Jong van Lier, 2021). Furthermore, the choice of degree of water saturation as an indicator of water stress allows a straightforward and practical measure of the soil water stress status, as matric water potential, although it has a better and more well-established relationship with water flow and root water uptake, is more time-consuming, laborious, and requires technical expertise to determine, which may be an important limitation for some field studies. In addition, the degree of water saturation is inherently associated with soil mechanical impedance, as soil penetration resistance was described using the Busscher equation, which relates penetration resistance to soil bulk density and volumetric water content. Because volumetric water content is directly used to calculate the degree of water saturation, this indicator is indirectly embedded in the mechanical impedance adopted in this study, linking soil water status to mechanical strength constraints on root elongation. This approach facilitates simulations in environments where both water deficit and saturation) can be critical stress factors, making the model applicable to a wide range of hydric conditions. Although the empirical parameters used (X_o_, Y_o_, A, B and C; **Table 2**), which are not physical-based, comparable and should not be independently interpreted, the equation can be used into the process-based models and the results indicate that including the effect of soil structure in the model provides a more physically grounded representation of the soil–root interaction processes during root elongation. Addtionally, the large difference in the Gaussian parameter *a* between Field and Packed samples results from differences in the variability and functional relatioship between observed root elongation responses and stress combinations under each structural condition. Our model, although semi-empirical, was able to highlight the essential soil structure role in mitigating physical stress on soybean root elongation process, suggesting the model generation and such a parameterization should be oriented by the soil structure. However, since the parametrization was structure-oriented, the extrapolation of fitted parameters under other structural conditions may lead to a reduction in the model predictive capacity, being important the equation calibrating according to the soil structural conditions. In addition, although a wide range of soil penetration resistance (0.5 MPa ≤ Q_p_ ≤ 4 MPa) and degree of soil water saturation (50% ≤ S ≤ 90%), root elongation under fully saturated and drier soil conditions, associated with greater mechanical impedance, was not explored. This introduces greater uncertainty in the model under these conditions, which are expected to be more restrictive to soybean root elongation. Furthermore, as the impact of physical stress and structural conditions on root elongation was assessed only at an early stage of crop development, longer-term evaluation of these soil–root interactions are still required to verify whether these relationships are conserved over time. Regarding the prediction of soil penetration resistance, Gao et al. (2016b) previously reported the important effects of bulk density and water status, represented by soil water matric potential and degree of water saturation, on soil penetration resistance for root elongation (Bengough et al., 2011), supporting the strong dependence of mechanical impedance on these variables, as highlighted in our study. Notwithstanding, despite the comprehensive fitting of the Busscher model and its satisfactory performance in representing the effects of bulk density and water content on soil penetration resistance, soil strength may vary along the soil profile under the same bulk density and water status (Gao et al., 2016b), resulting in different levels of mechanical impedance for root elongation.

Soybean root elongation was greater when grown under Field soil structure compared with packed-soil conditions, with pronounced differences observed under high physical constraint conditions, particularly in scenarios of high soil penetration resistance (e. g., **Figure 4**). In the same experimental site, Pereira et al. (2025) reported the greater soybean rooting deep in well-structured soil under no-tillage system, what is related with the highest soybean yield, which was emphasized by Balbinoti Junior et al. (2024), supporting our results and model applicability. The critical soil penetration resistance that limits root system growth is frequently reported to range between 2.0 (Bengough et al., 2011), 2.5 MPa (Taylor et al., 1966) up to 3.5 MPa (Moraes et al., 2014). However, depending on the species and water conditions, roots may withstand higher values. The literature already shown root elongation under higher soil penetration resistances, for example 4 MPa for sugarcane (Grande et al., 2025), since that adequate soil water availability is maintained (Bengough et al., 2011). In our study, the increase in soil penetration resistance under soil water matric potential of -60 hPa (equivalent to nearly 70% degree of water saturation) had a slightly more pronounced effect on soybean root elongation in Packed structure, attaining a decrease of root elongation of almost 74% when grown in Packed structure and 52% in Field structure. This behaviors indicates that, in addition to water availability, soil structure plays an additional role in mitigating the effects of mechanical stress on root growth.

During root growth, particularly under mechanical limitations, roots tend to grow in cracks or macropores, which offer low-resistance channels, likely present in the Field soil samples in our study. This may lead to heterogeneous root distributions throughout the soil profile (van Noordwijk et al., 1993). Furthermore, when growing in these pore spaces (i.e. macropores or biopores), roots are often only partially in contact with the soil matrix rather than being fully surrounded by soil, as assumed in many classical uptake models. This partial root–soil contact alters the effective root–soil interactions, which was not considered in our study and may imply complex structure-affected root growth under physical stress conditions, especially hydric stress (Willigen et al., 2018). Additionally, although greater root elongation was observed as soil bulk density decreased (**Figure 2**) across the structural conditions, previous approaches such as thin-section techniques have demonstrated that loose soils may lead to reduced soil–root contact (Veen et al., 1992), altering the optimum water status range and, consequently, the parameterisation.

The soil physical stress markedly affected the root growth, implying in a decrease in root area surface under constraining physical conditions, especially associated with high mechanical impedance (**Figure 3**). However, our study emphasized important differences between root diameter classes, which was controlled by the combined effects of mechanical impedance and soil water stress. The strong reductions in fine root length observed with increasing soil penetration resistance, particularly in the Packed structure (up to 93%), indicate the high susceptibility of smaller diameter roots to soil physical constraints (**Figure 4**). Fine roots exert lower axial growth pressure and therefore depend more strongly on pores and structural pathways of lower mechanical resistance to explore the soil (Bengough et al., 2011; Moraes et al., 2018). In soils with a Packed structure with high mechanical impedance, the low-resistance pathways, offered by the pore space, become scarce, limiting the fine roots elongation and markedly their length (Giuliani et al., 2024).

In contrast, thicker roots showed greater tolerance to increasing soil penetration resistance, which explains the smaller reductions observed in this diameter class. Roots with larger diameters are less prone to bending when encountering zones of high mechanical impedance and are therefore better able to penetrate resistant soil layers (Bengough et al., 2011). In addition, mechanical stress can induce radial thickening of roots, increasing the proportion of coarse roots while reducing the production of fine roots as a strategy to alleviate mechanical stress at the root tip (Lin et al., 2016). Soil water stress also played a key role in regulating root elongation, particularly for fine roots, also due its indirect effect on soil penetration resistance (**Figure 4**). Under water deficit conditions (-5000 hPa), reductions of more than 50% in fine root length were observed in both soil structures. Soil drying increases soil penetration resistance exponentially, intensifying the mechanical constraint imposed on root growth (Whitmore and Whalley, 2009; Passioura, 2002) and further restricting the elongation of roots with smaller diameters (Materechera et al., 1991). This interaction between mechanical impedance and soil water status has been widely recognized as a major factor controlling root growth in structured soils.

Under near-saturated conditions (-10 hPa), the greater root length observed in thicker roots may also be associated with plant adaptations to limited oxygen availability. In poorly aerated soils, plants can modify the morphology and anatomy of their roots, including radial thickening and the formation of aerenchyma tissues (Striker et al., 2007), thus facilitating the transport of oxygen from the aerial parts to the root tips in growing plants (Lipiec et al., 2012; Striker et al., 2007). Although the anatomical characteristics of the roots were not measured directly in this study, we believe that such mechanisms contributed to maintaining root elongation under gaseous stress conditions.

The comparison between Field and Packed soils across different levels of penetration resistance shows that Field soil structure provides a low-stress condition for soybean root elongation, especially when water availability is a limiting factor (e. g., **Figure 6**). The greater soybean root elongation when grown in preserved soil structure (i. e., Field) (**Figure 4a**) can be explained by the likely presence of continuous and interconnected pore networks (i.e., biopores) offer low mechanical impediment pathways for root elongation (Wendel et al., 2022), markedly across compacted soil layers, allowing the deep root growth and water uptake in soil subsurface (Thorup-Kristensen et al., 2020). These pores are formed by continuous root activity and become accessible to subsequent plants as the roots decay (Lucas et al., 2022). In conservation systems based on the absence of soil disturbance (i.e. no-tillage), the presence of cover crops contributes to the maintenance and formation of a continuous and interconnected pore network (Zhang and Peng, 2021), which plays an important role in key physical processes such as water flow (Zhang et al., 2022), gas diffusivity (Talukder et al., 2022), as well as offering low-impediment pathways for root elongation (Islam et al., 2024; Behrend et al., 2025). Our findings support the role of soil structure, especially as soil physical stress increases (i.e., high mechanical impedance associated with near-saturated or dry soil conditions). For instance, although relative root elongation in Packed soil was greater than in Field soil under loose soil conditions (**Figure 4b**), root elongation was up to 62% higher in field soil as physical soil stress became markedly constrained (**Figure 4a**). Furthermore, Gao et al. (2016a) emphasized that soil structure should be considered when investigating root–soil interactions (Jin et al., 2013), rather than focusing solely on physical stress levels, which aligns with our results.

Our study showed that under conditions of low soil penetration resistance (0.6 MPa), the Packed structure exhibited a higher relative root elongation than the Field soil structure. Nevertheless, this was observed only under an optimal hydric condition, with a greater soybean relative root elongation when grown in Field soil structure under dried-soil (degree of water saturation of ~ 50%) and near-saturation (Saturation degree > 87%) condition, especially as the degree of soil compactness and penetration resistance increased. Under near-saturation conditions, excess water fills soil pores, restricting oxygen diffusion, and leading to hypoxic conditions (O_2_ deprivation) (Daniel and Hartman, 2024). For sensitive plants such as the soybean, which lack complex aeration adaptations such as aerenchyma, soil gas diffusivity is fundamental to plant growth (Garcia-Vila et al., 2025). Our results showed more tolerance of the soybean cultivated in field soil structure with continuous pores from no-tillage than in packed soil structures (**Figure 2a**, **Figure 6**). Hypoxic stress is also associated with increased production of reactive oxygen species at high concentrations (Mehra et al., 2025), as well as with changes in redox potential within root cells, leading to alterations in root development and inhibition of apical meristem activity (Kumar et al., 2020). The root growth into soil biopores aliviated mechanical impedance (Jin et al., 2013), in ours results we shown that structured soil under no-tillage less impacts of hypoxic-related stress, due to better distribution and connectivity of macropores that facilitate gas exchange. The presence of an interconnected and continuous pore network, arising from no-tillage systems (Talukder et al., 2022), provides an important pathway for soil gas diffusivity (Fu et al., 2024), particularly under waterlogged conditions and compacted soils. Accordingly, we observed greater soybean root elongation when grown in Field soil structure under near-saturation conditions and at bulk density of 1.18 Mg m^-3^, which can be explained by the contribution of soil structure to the mitigation of hypoxic stress. However, as the bulk density increased to 1.30 Mg m^-3^ (**Figure 2a**), no differences were observed between structural conditions, indicating that the higher level of soil compaction imposed a strong physical constraint on root elongation, even when grown in Field soil structure.

As observed under near-saturation conditions (**Figure 2a**), Field soil structure exhibited greater root elongation than Packed structure under water deficit conditions (**Figures 2d, e**), particularly at a soil water matric potential of -1000 hPa (e. g., **Figure 2d**). This difference between treatments became more pronounced as soil penetration resistance increased, highlighting the combined effect of water availability and mechanical impedance on the relative root elongation (**Figure 6**). The greater soybean root elongation observed under dried soil conditions in soil with a preserved structure may be explained by the contribution of the pore space to enhanced soil–root contact (Xiong et al., 2022) supporting radial hydraulic conductivity at this interface, as well as facilitating root penetration through layers with greater mechanical impedance (Giuliani et al., 2024), mainly under higher bulk density (i.e., bulk density > 1.30 Mg m^-3^). Notwithstanding, drier soils, observed at a soil water matric potential of -5000 hPa (**Figure 2e**), exhibited high mechanical and hydraulic impedance, markedly reducing root elongation under both structural conditions. Therefore, although the soil structure plays a fundamental role in mitigating physical stresses and supporting root growth, its buffering capacity may be severely constrained under extreme soil physical stress conditions. This response may be associated with the soybean sensitivity to such stresses, highlighting the importance of parameterizing other plant species.

We observed that the combination of mechanical and hydric stress resulted in a reduction in the soybean root elongation. However, this reduction occurred more gradually in Field soil structure. Moraes and Gusmão (2021) described that soybean root elongation were reduced with increases of penetration resistance, especially under severe water stress conditions, indicating that both water limitation and soil mechanical impedance are critical factors for root growth, supporting our findings. However, the soil structure can be a primary limiting factor for root elongation. Valentine et al. (2012) demonstrated that root elongation is strongly related to soil physical properties, particularly pore arrangement and volume, which allow the maintenance of high root growth rates even under increased soil mechanical impedance. Similar mechanisms have been reported for maize root growth in Vertisols under the use of cover crops (Zhang et al., 2022). Modeling studies have further confirmed that soils with the presence of biopores and continuous pore networks increase rooting depth and total root length, translating into a significant mitigation of transpiration deficits during drought periods (Landl et al., 2019). The variation in relative root elongation in response to different levels of soil penetration resistance and soil water saturation highlights the complex interaction between mechanical impedance and water availability. This understanding is crucial for the development of management practices that optimize the root environment and enhance crop growth.

The results presented in this simple model for soybean root elongation can improve the models to predict the root growth in field condition, contributing to simulation models that account for the effects of soil structure, such as biopores and continuous pores (Mulazzani et al., 2022). Incorporating these structural factors is crucial for a more accurate representation of root growth dynamics and water and nutrient uptake (Giuliani et al., 2024). Models that disregard soil structure tend to oversimplify soil–plant interactions over time, resulting in less realistic predictions of crop performance (König et al., 2023), particularly under mechanical and water stress conditions. By integrating the effect of soil structure into the model, it becomes possible to highlight the complexity of interactions within the soil–plant system. As a result, simulations become more robust, providing relevant insights for agricultural planning and practices, helping farmers to maximize productivity while minimizing the impacts of climate change (Jarvis et al., 2024). Although this study has significantly contributed to the understanding of the interaction between soil structure and root growth in grain-producing crops, there remains a need to expand the parameterization to include other crops, especially cover crops that are widely used to improve soil physical quality and functions (Gentsch et al., 2024; Bertollo et al., 2021). Modeling these complex interactions for both grain and cover crops would represent a significant advancement in this scientific field.

## Conclusion

Under the studied conditions (0.5 MPa ≤ Q_p_ ≤ 4 MPa and 50% ≤ S ≤ 90%), in an Oxisol with very clayey texture, the effect of soil structure of no-tillage system was used to parameterize the soybean root elongation based on relationship with mechanical (soil penetration resistance) and hydric (degree of water saturation) stresses. This approach resulted in the development of semi-empirical models for both Field and Packed soil structures. Soil structure plays an essential role in mitigating mechanical and hydric stresses, highlighting its importance in soil–root interactions.

## Data Availability

The raw data supporting the conclusions of this article will be made available by the authors, without undue reservation.
